# Erythropoietin and its derivatives: from tissue protection to immune regulation

**DOI:** 10.1038/s41419-020-2276-8

**Published:** 2020-02-03

**Authors:** Bo Peng, Gangcheng Kong, Cheng Yang, Yingzi Ming

**Affiliations:** 1 Transplantation Center, The Third Xiangya Hospital, Central South University, Changsha, Hunan 410013 PR China; 2 Department of Urology, Zhongshan Hospital, Fudan University, Shanghai Key Laboratory of Organ Transplantation, Shanghai, 200032 PR China; 3 Zhangjiang Institute of Fudan University, Shanghai, 201203 PR China

**Keywords:** Cell death, Immunology, Drug development

## Abstract

Erythropoietin (EPO) is an evolutionarily conserved hormone well documented for its erythropoietic role via binding the homodimeric EPO receptor (EPOR)_2_. In past decades, evidence has proved that EPO acts far beyond erythropoiesis. By binding the tissue-protective receptor (TPR), EPO suppresses proinflammatory cytokines, protects cells from apoptosis and promotes wound healing. Very recently, new data revealed that TPR is widely expressed on a variety of immune cells, and EPO could directly modulate their activation, differentiation and function. Notably, nonerythropoietic EPO derivatives, which mimic the structure of helix B within EPO, specifically bind TPR and show great potency in tissue protection and immune regulation. These small peptides prevent the cardiovascular side effects of EPO and are promising as clinical drugs. This review briefly introduces the receptors and tissue-protective effects of EPO and its derivatives and highlights their immunomodulatory functions and application prospects.

## Facts

- In addition to erythropoiesis, erythropoietin (EPO) can bind the tissue-protective receptor (TPR, namely EPOR/CD131 heterodimer) and plays an important role in tissue protection and immune regulation.
- EPO can be induced and secreted by both parenchyma cells and immune cells under hypoxia, and the locally produced EPO functions in a paracrine-autocrine manner.
- Both innate and adaptive immune cells express TPR, and they can be directly regulated by EPO.
- The nonerythropoietic EPO derivatives mimic the structure of EPO and eliminate the side effects, which are promising in clinical application.

## Open questions

- Why do the effects of EPO and its derivatives on immune cells show discrepancy, especially between in vitro and in vivo studies?
- Is there a better nonerythropoietic EPO derivative that has a stronger affinity for TPR?
- Is it possible to develop oral preparations for alternative administration routes of the small peptide EPO derivatives?
- Besides immune cells, TPR-mediated signal has significant impacts on other cells, for instance, renal tubular epithelial cells. It is very important and interesting to explore the effect of TPR pathway on other types of cells.

## Erythropoietin, not just erythropoiesis

Erythropoietin (EPO), an evolutionarily conserved hormone mainly produced in the kidney, has been well documented for its indispensable role in erythropoiesis. EPO belongs to the type 1 cytokine superfamily and has 165 amino acids forming four α helices^1^. In humans, the plasma half-life of kidney-produced EPO is 5–6 h due to high levels of glycosylation. When erythrocyte levels decline, the renal tubular interstitial cells detect relative hypoxia and secrete EPO into the circulation in a classic endocrine manner. Then, EPO migrates to bone marrow, binds the homodimeric EPO receptor (EPOR)_2_ on the erythroid progenitors, and promotes erythropoiesis. Due to the high affinity of (EPOR)_2_, the trace amounts of EPO in human serum regulated by a classic negative feedback loop are able to maintain the homeostasis of erythropoiesis^2^.

In recent years, numerous studies have shown that EPO acts far beyond erythropoiesis. In hypoxia, trauma or inflammation, many tissues produce EPO at the borders surrounding injury sites; EPO plays central roles in tissue protection and restoration. Previously, these effects were believed to be mediated by the inhibition of proinflammatory cytokines and the downregulation of apoptosis^1,3^. However, recent studies have revealed that EPO and its derivatives could also directly work on the immune cells. In this review, we briefly reviewed the receptors and tissue-protective effects of EPO and the development of its nonerythropoietic derivatives. We further highlight the immunomodulatory functions and application prospects of EPO in the clinic.

## What is the tissue-protective receptor (TPR)?

A remarkable characteristic of the type 1 cytokine superfamily receptors is that they are commonly composed of different subunits. The β common receptor (βCR), or CD131, is the subunit receptor shared by type 1 cytokines, including granulocyte-macrophage colony-stimulating factor (GM-CSF), interleukin (IL)-3 and IL-5^4^. Through affinity chromatography and coimmunoprecipitation, βCR and EPOR were shown to covalently bind and form a heteromeric complex. Immunocytochemistry further showed that these two subunits colocalize^5^. Notably, the tissue-protective and healing effects of EPO and its derivatives were abolished in the absence of βCR^5–7^. Some EPO derivatives, including carbamylate EPO (CEPO), helix B surface peptide (HBSP) and cyclic helix B peptide (CHBP), did not bind (EPOR)_2_ and were not erythropoietic but still showed tissue-protective effects^8–10^. These studies revealed that the tissue-protective effects of EPO and its derivatives are mainly mediated by the heterodimer of EPOR/βCR; thus, this heterodimer was called tissue-protective receptor (TPR) or innate repair receptor (IRR). Recently, the receptor for vascular endothelial growth factor (VEGFR2) was also reported to be involved in the composition of TPR and is induced in hypoxia and plays a role in nitric oxide (NO) production in endothelial cells^11^.

The emergence of nonerythropoietic EPO derivatives makes them available for illustrating the signaling pathways of TPR. When ligands bind TPR, multiple pathways are activated and overlap some in erythropoiesis^1,12,13^. The initial step is the autophosphorylation of Janus kinase 2 (Jak2), which then activates three main cascades. The first cascade is the signal transducer and activator of transcription (STAT) pathway, which includes STAT3 and STAT5, leading to upregulated survival signals and apoptotic resistance like in erythropoiesis^1,14^. The second cascade involves the phosphatidylinositol 3-kinase (PI3K) and Akt pathway. The PI3K/Akt pathway phosphorylates glycogen synthase kinase 3β (GSK3β), significantly decreasing its activity, inhibiting mitochondrial permeability transition (MPT) and stabilizing mitochondria, leading to the inhibition of apoptosis^15^. The inhibition of GSK3β also downregulates nuclear factor-κB (NF-κB), thus reducing inflammation and edema^6,16^. The third cascade is the mitogen-activated protein kinase (MAPK) pathway, which also inhibits GSK3β and attenuates inflammation^14,16^. In addition, the PI3K/Akt pathway promotes the production of NO via the activation of endothelial nitric oxide synthase (eNOS), which increases blood flow, attenuates regional injury and induces endothelial cell proliferation, migration and healing^6,16,17^. The AMP-activated protein kinase (AMPK) pathway, which is downstream of βCR, was also reported to induce eNOS and NO production after EPO stimulation^18^.

## TPR-mediated tissue-protective effects

When tissue suffers from pathogen invasion, trauma or hypoxia, a highly orchestrated defense program is triggered, characterized by the production of proinflammatory cytokines and chemokines. These molecules recruit circulating immune cells to destroy pathogens and remove damaged cells. They also cause vascular thrombosis and edema, which helps isolate damage but aggravates hypoxia^3^. Notably, the process is self-amplifying, causing necrosis and apoptosis via a positive feedback loop, which may lead to catastrophic injury to adjacent and distant tissues^1^. To confine the damage, protective and anti-inflammatory process occurs just following injury.

EPO is an important regulatory factor that helps maintain immune homeostasis. In most quiescent cells, EPOR and βCR are typically localized within intracellular compartments, but hypoxic and proinflammatory cytokines can induce the rapid translocation and expression of EPOR and βCR on cell surfaces; this process occurs earlier than the synthesis of EPO^19^. Hypoxia also induces the expression of hypoxia-inducible factor (HIF), which binds *EPO* enhancer (E-3′) and leads to the production of EPO^20^. In contrast, proinflammatory cytokines, such as tumor necrosis factor (TNF)-α, inhibit EPO production^21^. As a result, although cells at the central core of injuries express TPR, they lack the appropriate corresponding binding ligand due to high concentrations of proinflammatory cytokines and eventually die. At the periphery of injuries, EPO can be synthesized due to the relatively low levels of proinflammatory cytokines. Locally produced EPO diffuses inward and binds TPR, which inhibits inflammation, rescues cells and halts spread of injury. The antagonism of these molecules determines the scale of the injury^1,3^.

Unlike the high-affinity receptor (EPOR)_2_, TPR has a much lower affinity for EPO. To initiate the tissue-protective effects of TPR, the required EPO concentration is much higher than that in circulating serum^12^. Locally produced EPO is poorly sialated (hyposialated EPO; hsEPO), has a much shorter plasma half-life, and functions in a paracrine-autocrine manner^1^. Researchers have reported that totally enzymatically desialated EPO (asialo-EPO) has a half-life of only 1.4 min but still showed fully protective effects without erythropoiesis^22^. Therefore, in the microenvironment of an injury, locally produced hsEPO reaches high concentrations sufficient to activate TPR but does not influence erythropoiesis.

## Do EPOR- and TPR-mediated signals directly influence immune cells?

(EPOR)_2_ and TPR are expressed on a variety of immune cells, such as macrophages, dendritic cells, mast cells and lymphocytes^23^. An increasing body of evidence demonstrates that EPO and its derivatives can directly affect the manner by which immune cells exert their immunoregulatory effects.

### Innate immune system

#### Macrophage

**Fig. 1:**
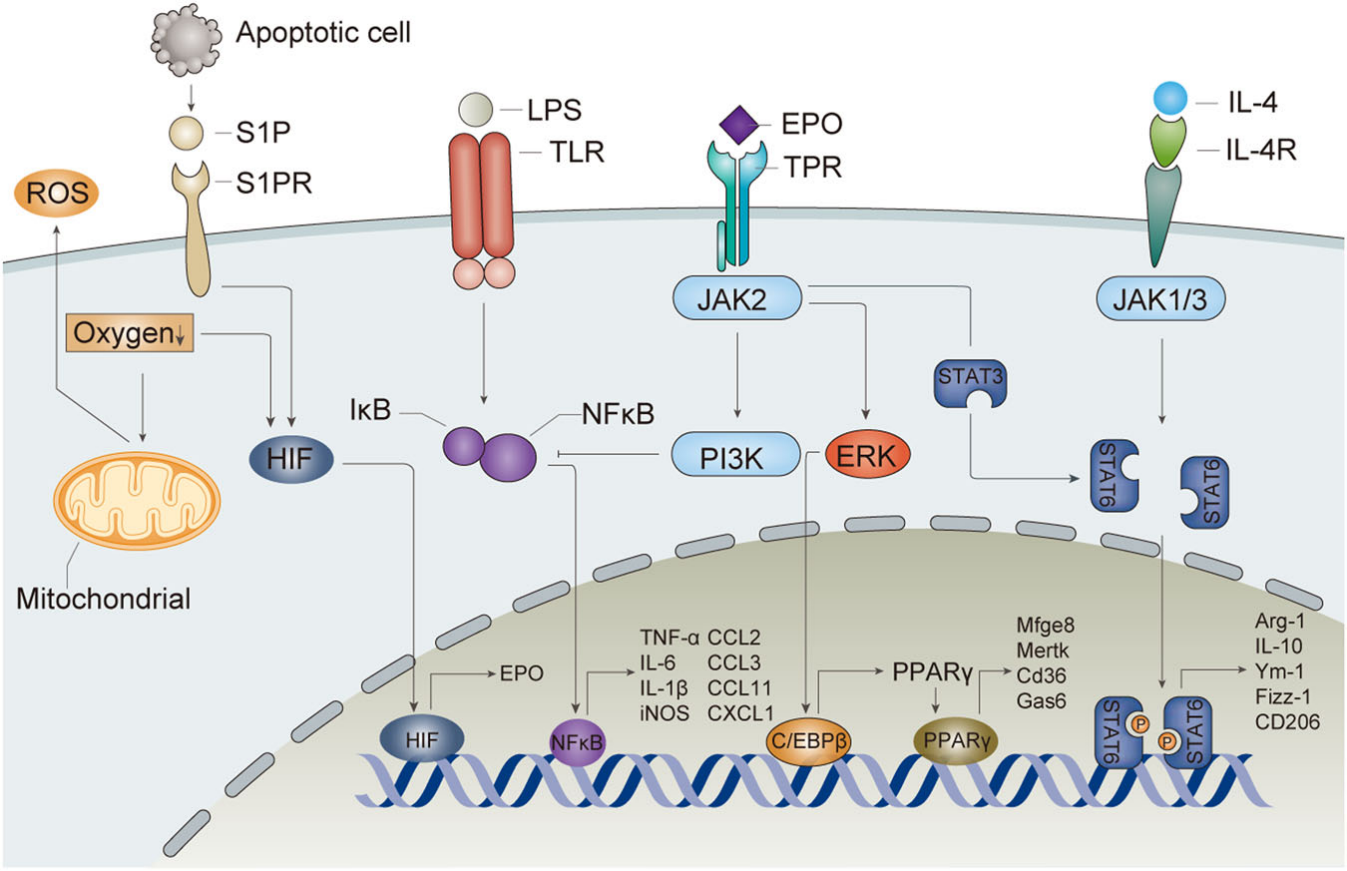
The effects of EPO on macrophages. EPO regulates the polarization of macrophages via shifting them to M2 phenotype and exerts anti-inflammatory effects. Activation of TLR on macrophages leads to upregulation of inflammatory mediators and polarization to M1 phenotype, which aggravates tissue injury. EPO shifts macrophages to M2 phenotype via EPOR/Jak2/STAT3/STAT6 signaling pathway in the presence of IL-4. Meanwhile, EPO inhibits NF-κB p65 activation via EPOR/Jak2/PI3K pathway. EPO also plays a vital role in clearing apoptotic cells and cell debris. S1P released from apoptotic cells and hypoxia upregulate EPO and EPOR expression via HIF complex. EPO signaling increases the levels of phagocytic receptors through PPARγ pathway and facilitates phagocytosis of apoptotic cells. EPO erythropoietin, TPR tissue-protective receptor, S1P sphingosine 1-phosphate, HIF hypoxia-inducible factor.

Macrophages play an important role in innate immunity and are the main source of proinflammatory cytokines. Researchers showed that macrophages expressed TPR at baseline and that EPO treatment significantly reduced TNF-α, IL-6 and inducible nitric oxide synthase (iNOS) expression by blocking NF-κB p65 activation^24^. EPO treatment led to reduced pathogen clearance in *Salmonella* infection and, in contrast, the amelioration of disease severity in experimental colitis^24^. Our group also showed that EPO suppressed the production of NO, TNF-α, IL-6 and IL-1β in dose-dependent manners in macrophages (Fig. 1)^25^.

Chemokines are important to the migration and recruitment of macrophages and other immune-competent cells. Studies have shown that EPO can directly influence the expression of chemokines by macrophages and modulate their migration. The production of C-C motif chemokine ligand 2 (CCL2) by macrophages relies on the stimulation of toll-like receptor (TLR) and the activation of the MyD88/NF-κB pathway. When TPR is activated, the downstream Jak2-PI3K/Akt pathway can suppress the expression of CCL2 by macrophages^24,26,27^. In vitro, EPO could decrease the levels of CCL2, CCL3, CCL11 and C-X-C motif chemokine ligand 1(CXCL1) expressed by macrophages and monocytes (Fig. 1)^27^. In vivo, similar results have been verified in islets transplant model^26^, experimental colitis mice^27^, pristane-induced systemic lupus erythematosus (SLE) mice^28^ and acute kidney injury mice^25^. These results indicate that EPO reduces the prolonged infiltration of inflammatory macrophages. However, contradictory phenomena were observed on resident macrophages. EPO can facilitate CCL2 production by Kupffer cells and promote the recruitment of monocytes to injured liver^29^. Similarly, increased levels of EPO can recruit more macrophages to laser-injured choroids^30^. This may be related to EPO-induced anti-apoptosis and proliferation effects after injury.

Macrophages can be induced to the classically activated M1 phenotype or the alternatively activated M2 phenotype. EPO and its derivatives can directly affect the polarization of macrophages and tend to shift macrophages toward the M2 phenotype to exert anti-inflammatory function and promote tissue healing (Fig. 1). Our group found that EPO ameliorated acute kidney injury by reducing macrophage infiltration and promoting M2 phenotype polarization in vivo^25^. CD206^+^ M2 macrophages and mRNA of M2 markers, including arginase-1, Ym-1, Fizz-1 and CD206, were significantly increased in the EPO-treated group. In vitro, although EPO suppressed proinflammatory cytokines secreted by M1 macrophages, EPO promoted M2 polarization only in the presence of IL-4. The EPO signaling pathway collaborated with the downstream pathway of IL-4 to promote M2 polarization; a possible mechanism may be through the Jak2/STAT3/STAT6 pathway^25^.

Efferocytosis is a process by which dead cells are cleared without eliciting unwanted immune responses^31^. Macrophages are the main phagocytic cells, but their regulation is poorly understood. Recently, EPO signaling has been reported to facilitate macrophages to clear apoptotic cells and cell debris, thus promoting immune tolerance (Fig. 1). In self-limited peritoneal inflammation mice, EPO concentration in peritoneal fluid appeared bimodal, corresponding with the infiltration of neutrophils and macrophages accompanied with hypoxia caused by respiratory burst^32^. In contrast, the lack of detectable EPO in situ or the macrophage-specific EPOR knockout mice yielded chronic inflammation^32^. Meanwhile, apoptotic cells released the “find me” signal sphingosine 1-phosphate (S1P), which specifically binds to S1P receptor 1 (S1PR_1_) and enhances the expressions of HIF-1α and EPO in macrophages. EPO further induced EPOR and activated the EPOR-Jak2-ERK-C/EBPβ-peroxisome proliferator-activated receptor-γ (PPARγ) signal, increased the expression of *Mfge8*, *Mertk*, *Cd36* and *Gas6*, and promoted apoptotic cell clearance^33^. Through effective efferocytosis, apoptotic cells can be rapidly eliminated thereby preventing a triggering of the immune system. In this way, efferocytosis promotes immune tolerance and tissue restoration (Fig. 1).

**Fig. 2:**
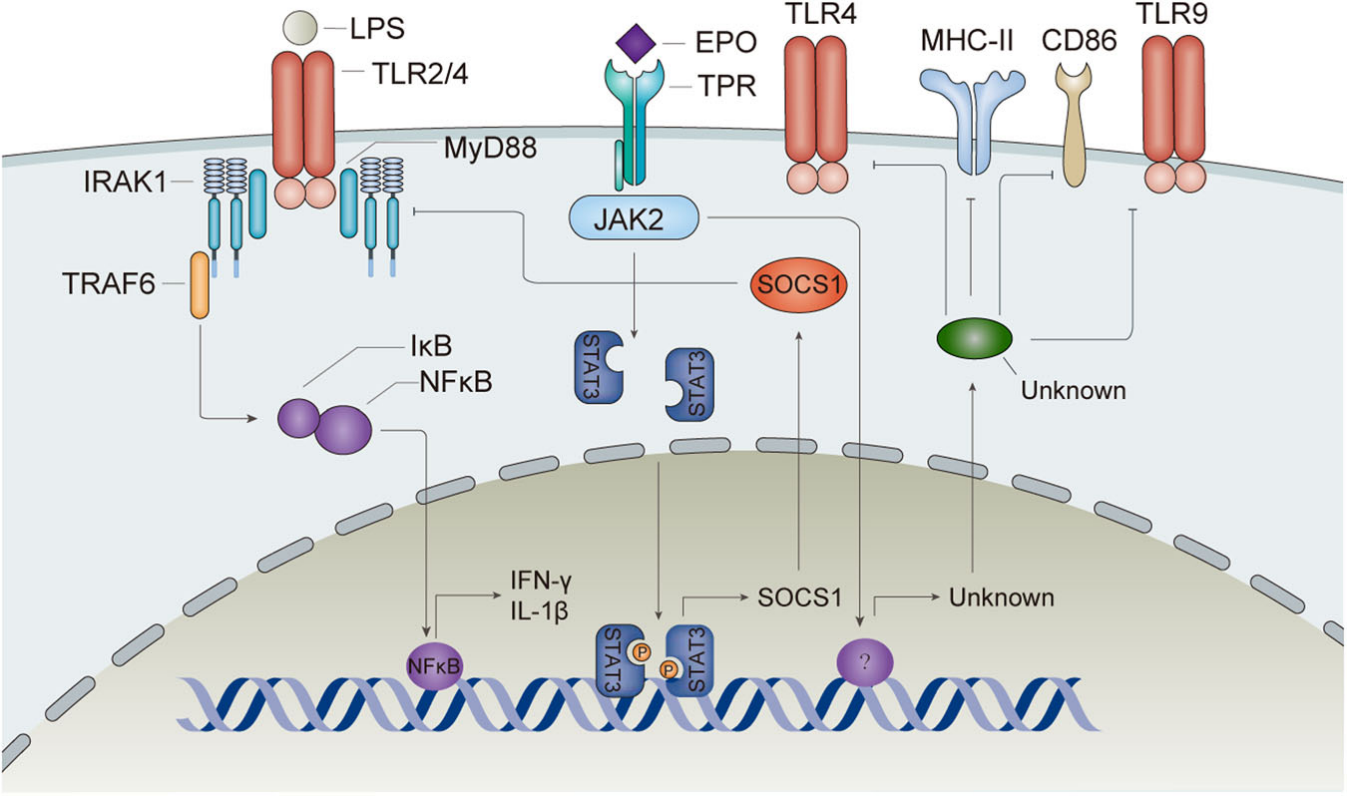
EPO suppresses the maturation of dendritic cells. EPO binds TPR and then activates Jak2/STAT-3/SOCS1 pathway. SOCS1 inhibits LPS signaling cascade. In addition, EPO can decrease the expression of MHC-II and costimulatory molecules. EPO erythropoietin, TPR tissue-protective receptor, HO-1 heme oxygenase-1, MHC-II major histocompatibility complex class II.

#### Dendritic cells

Dendritic cells (DCs) play a central role in antigen presentation and initiating adaptive immunity. In response to different stimuli, DCs show great plasticity and maintain homeostasis between protective immunity and tolerance^34^. EPOR is expressed on DCs, suggesting they are EPO targets^35–37^. Our group demonstrated that CHBP could ameliorate acute rejection (AR) in a rat kidney transplantation model via inhibition of DC maturation^38^. Rats treated with CHBP showed lower levels of IL-1β and IFN-γ but higher levels of IL-4 and IL-10 in serum and in renal allografts. The expression of major histocompatibility complex class II (MHC-II) or CD86, which are markers for mature DCs, decreased on DCs in renal allografts treated with CHBP. An in vitro study of bone marrow-derived DCs showed similar results, and the function of CHBP-treated DCs to induce the T-cell proliferation was significantly inhibited. The possible mechanism was the activation of Jak2/STAT3/suppressor of cytokine signaling 1 (SOCS1) pathway, where SOCS1 inhibited the TLR2/4 signaling pathway (Fig. 2)^38^. In experimental cerebral malaria mice, the analysis of DCs from the spleen showed that recombinant human EPO (rhEPO) inhibited the maturation and activation of DCs with decreasing levels of MHC-II, CD86, TLR4 and TLR9 (Fig. 2)^39^. However, the specific mechanism requires further exploration.

In contrast, researchers have also found that EPO could promote the maturation of DCs and enhance their immunostimulatory ability^35–37^. EPO was reported to enhance antigen uptake and promote the maturation of immature monocyte-derived DCs (MoDCs). In addition, EPO treatment upregulated the expressions of MHC-II, CD80, and CD86 on immature DCs; this effect was absent on mature DCs in the spleen^37^. Such effects may be associated with the activation of Akt, MAPK, and NF-κB and Tyr-phosphorylation in the STAT3 signaling pathway^36^. However, these results were observed from in vitro studies or mice without disease model, and EPO concentrations and in vitro stimulation schedules varied. These contradictory EPO effects on DCs require additional study.

#### Mast cells

Mast cells protect against parasitic infection and anaphylactic reaction via releasing secretory granules and activating type 2 immune responses^40^. Recently, mast cells have been shown to express EPOR, but they have quite different characteristics. Mast cells highly express intracellular EPOR; however, on cell surface, the basal EPOR expression is low and only a small proportion (~20%) of mast cells express EPOR at high levels^41^. One possible reason may be the inefficiency of the transport system^41^, and another may be the specific structure of the intracellular EPOR^42^. The intracellular EPOR of mast cells is soluble and located in the secretory granules, being incomplete with a 43 kDa extracellular domain and lacking the intracellular domain compared with the typical EPOR^42^. Interestingly, neither the typical EPOR signaling pathway nor the TPR is activated in mast cells; instead, the mast cell marker CD117, or c-kit, is involved in EPOR signaling^41^. In vitro, EPO decreases mast cell secretions of IL-6 and TNF-α after stimulation by LPS through the activation of the EPOR/c-kit complex^41^. Another study showed that the administration of EPO regulated mast cells activities and ameliorated injury caused by overactive immune responses triggered by mast cells^43^. However, many questions remain. Cellular trafficking mechanisms of soluble EPOR in secretory granules, soluble EPOR functions, downstream EPOR/c-kit pathways and EPO crosstalk with other immune cells require further study.

### Adaptive immune system

#### T cells

Previously, it was believed that lymphocytes did not express EPOR; consequently, EPO was considered to have no direct effect on lymphocytes^44^. However, recent findings challenged this concept^45^. rhEPO is routinely used to correct anemia in hemodialysis (HD) patients. As such, Lisowska et al. performed a study to explore the effects of rhEPO on CD4^+^ lymphocytes from HD patients and found that rhEPO normalized the proliferative ability and activation markers of CD4^+^ lymphocytes, including CD28 and CD69^46^. Similar results were obtained in the myelodysplastic syndromes (MDS) patients whose immune system was impaired^47^. Through quantitative flow cytometry and reverse transcription-polymerase chain reaction (RT-PCR), researchers confirmed the existence of EPOR on human T and B lymphocytes^23^. Interestingly, they found that CD4^+^ lymphocytes from HD patients treated with rhEPO expressed higher levels of EPOR than those without rhEPO treatment^23^. However, the in vitro study showed the opposite observation, wherein peripheral lymphocytes and monocytes preincubated with rhEPO had decreased EPOR expression^23^. The discrepancy might be caused by the complex internal environment of HD patients, whose immune system was already impaired^48^. Nevertheless, these findings confirmed the fact that T lymphocytes could express EPOR and thus they could be the targets of EPO.

**Fig. 3:**
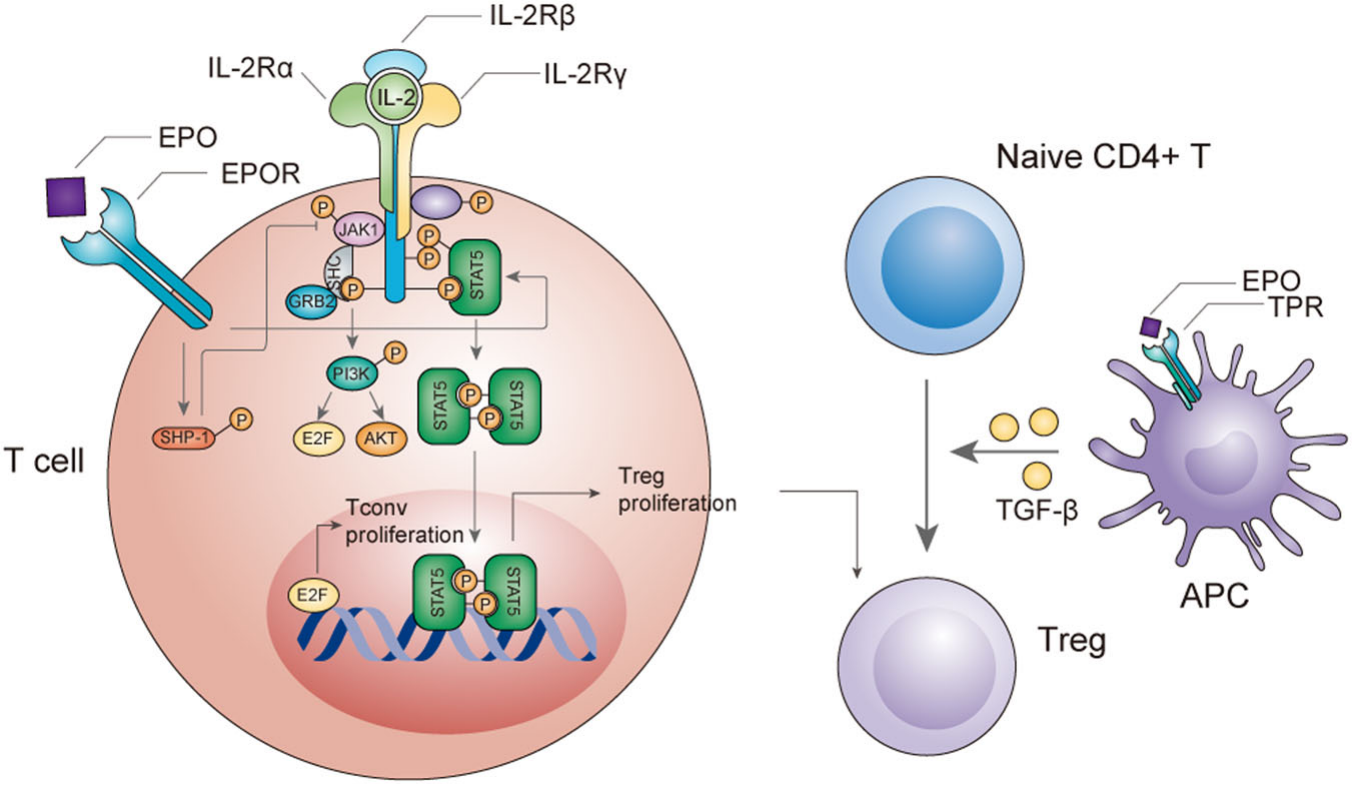
The effects of EPO on T cells. EPO directly promotes the proliferation of Treg but inhibits the expansion of Tconv through the molecular crosstalk with IL-2 pathway. EPO also upregulates TGF-β expression of APC via TPR, which induces Treg differentiation from naïve CD4 T cells. EPO erythropoietin, Treg regulatory T cells, Tconv conventional T cells, APC antigen-presenting cells, TPR tissue-protective receptor.

Very recently, a series of studies revealed that EPO had an immunoregulatory effect by acting on T cells^49–51^. EPO directly inhibited the proliferation of conventional T cells (Tconvs) in a dose-dependent manner without inducing apoptosis and conversely facilitated Treg proliferation^49,50^. The mechanism lay in the crosstalk between EPO signaling and the proliferative signaling of T cells. After IL-2/IL-2R ligation, the proliferation of Tconvs was mainly mediated by IL-2Rβ/Akt signaling, whereas in Tregs, pAkt was maintained at a low level and proliferation was mainly mediated by IL-2Rγ/STAT5 signaling. EPO induced the protein tyrosine phosphatase SHP-1, which uncoupled the IL-2Rβ/Akt signaling in Tconvs but had minimal impact in Tregs. In contrast, EPO enhanced IL-2Rγ/STAT5 signaling in Tregs and promoted their proliferation and stability (Fig. 3)^49,50^. Notably, this inhibitory effect of EPO was not mediated by the TPR, because ARA290, the nonerythropoietic derivative of EPO, did not show similar effects^49^.

Studies have also shown that EPO and its derivatives directly modulated T-cell differentiation. Under Th1 polarizing conditions, EPO diminished Th1 polarization but did not alter Th2 polarization or induce Tregs^49^. However, an in vivo study of experimental autoimmune neuritis (EAN) rats showed that the administration of ARA290 promoted the development of Th2 and Treg subsets but suppressed Th1 and Th17 subsets^52,53^. An analysis of draining lymph nodes revealed that after the ARA290 intervention, the master transcription factors of the Th2 and Treg subsets, GATA3 and Foxp3, respectively increased, whereas RORγt, the transcription factor of the Th17 subset, decreased^52,54^. The possible EPO mechanism that inhibited Th17 induction may be through the p38/SGK1-dependent pathway, which was confirmed in Th17-dependent autoimmune kidney disease models^51^.

In summary, T cells, especially activated T cells, express EPOR and are directly regulated by EPO. After ligation to EPOR or TPR, EPO and its derivatives exert direct immunoregulatory effects on T cells by modulating the function and differentiation of T cells.

#### B cells

Erythropoiesis, bone marrow microenvironments and B-lymphopoiesis are intimately associated with EPO signaling. Along with promoting erythrocyte proliferation, EPO induces a loss of trabecular bone volume to induce hematopoiesis and reduce the number of vessels in bone marrow^55^. At the same time, B cell development is impaired by EPO treatment due to significant reductions in pro-B and pre-B cells^55^. In humans, the short-term administration of rhEPO has shown significant decreases in B cells, mainly naïve B cells and IgD^−^CD27^−^ B cells in peripheral blood^56^. The suppressive effect of EPO on B cells may be related to changes in the bone marrow microenvironment or the direct ligation to EPOR on B cells^23^. However, the specific mechanism remains unknown.

### The EPOR and TPR signalings connect innate and adaptive immune systems

The activation and differentiation of T cells require the stimulation of antigen-presenting cells (APCs), which provide MHC-peptide complex with costimulatory molecules. The effects of EPO on APCs have been discussed above, and the EPO modulated APCs can further influence the adaptive immune system. Through binding TPR, EPO stimulated APCs, including monocytes, macrophages and kidney tubular cells, to express TGF-β, which converted naïve CD4^+^ T cells into functional Foxp3^+^Tregs (Fig. 3)^50,57^. In a controlled prospective cohort study, EPO administration was confirmed to augment peripheral CD4^+^CD25^+^CD127^lo^ Tregs in chronic kidney disease patients^50^. Similarly, EPO promoted T-cell suppression in a peritoneal cavity cell culture model which simulated a high myeloid to lymphoid cell ratio in the tumor microenvironment, but EPO showed no effects on spleen cells with normal myeloid to lymphoid cell ratios^58^. The mechanism was through the induction of iNOS from macrophages, which caused a disturbance in arginine catabolism^58^. These observed EPO effect discrepancies on Th2 polarization between in vivo and in vitro studies (which have been discussed above) may be attributed to the involvement of APCs.

## EPO biology in cancer

Cancer patients frequently suffer from cancer-associated chronic anemia and chemotherapy-induced anemia (CIA), which are associated with reduced quality of life and risk of hypoxia-induced treatment resistance^59,60^. As an effective erythropoiesis-stimulating agent (ESA) to correct anemia, EPO was found to improve quality of life and reduce the requirement of transfusions in early studies^61^. However, recent findings raise concerns about the use of EPO in cancer patients, which may be associated with tumor progression and poor patient prognosis. The systematic review from the Cochrane Library updated in 2012 reported that ESAs not only increased mortality during active study period (on-study mortality, hazard ratio (HR) 1.17, 95% confidence interval (CI) 1.06–1.29), but also decreased overall survival (HR 1.05, 95% CI 1.00–1.11) in cancer patients. The significant side effects of ESA treatment included thromboembolism, hypertension and thrombocytopenia/hemorrhage. However, the evidence of tumor progression was insufficient (risk ratio (RR) 1.02, 95% CI 0.98–1.06)^62^. The latest Agency for Healthcare Research and Quality (AHRQ) Comparative Effectiveness Reviews also reported increased on-study mortality (HR 1.17, 95% CI 1.04–1.31) for cancer patients receiving ESA treatment, but no discernible increase in mortality with long-term follow-up (HR 1.04, 95% CI 0.99–1.10). ESA treatment increased thromboembolism (RR 1.51, 95% CI 1.30–1.74), but notably, when ESA was used in patients with hemoglobin (Hb) less than 10 g/dL, fewer thromboembolic events and lower on-study mortality were observed^63^. Some meta-analyses dealing with specific cancers, such as lung cancer^64^, breast cancer^65^, lymphoproliferative malignancies^66^ and gynecological cancer^67^, reported controversial results on overall survival, but all of them showed reduced need of blood transfusions and insufficient evidence of tumor progression. Due to the potential risks, the 2019 American Society of Clinical Oncology (ASCO)/American Society of Hematology (ASH) guidelines on management of cancer-associated anemia suggested that ESA should only be used in patients with CIA whose cancer treatment was not curative in intent and whose Hb was less than 10 g/dL. ESA should not be offered to patients whose cancer treatment was curative in intent or most patients with nonchemotherapy-induced anemia^68^.

The preclinical researches make it possible for further exploration on EPO biology in cancer. Although the expression of CD131 on cancer cells remains unclear, the expression of EPOR has been verified both on mRNA and protein level^69–74^. Studies focus on EPOR signaling in cancer cells, which interacts with apoptotic pathway, hypoxia pathway and anticancer agents. EPOR signaling has been proved critical to tumor survival and proliferation. When EPOR signaling was blocked by EPOR knockdown or soluble EPOR against EPO, it inhibited tumor growth and invasion, and resulted in cell apoptosis^71,75–77^. Interestingly, in most studies, EPO stimulation had no significant effects on tumor proliferation, survival or invasion under normoxia^69,71–74,77,78^; only in few cases, such as in melanoma, EPO was reported to stimulate tumor growth both in vivo and in vitro on eIF4E-dependent pathway^79^. However, under hypoxia EPO significantly promoted cell proliferation^76,77^. One possible mechanism was that hypoxia not only induced the expression of EPOR, but also promoted translocation of EPOR from nucleus to cytoplasm and membrane, making it available to activate EPOR signaling^77^. In reality, the overgrowth of cancer cells frequently outstrips the supply of oxygen, leading to a hypoxia condition. During this process, EPO/EPOR pathway is believed to promote cancer progression.

Hypoxia also initiates angiogenesis, which greatly contributes to tumor growth, invasion and metastasis. Indeed, the vascular system and hematopoietic system develop from a common ontogenesis, which is named hemangioblast. As a result, a tight interplay exists between angiogenesis and hematopoiesis, and EPO plays an important role in angiogenesis^80^. Both EPOR and TPR were found on endothelial cells and endothelial progenitor cells (EPCs), and stimulation with EPO significantly promoted their proliferation, migration, tube formation and antiapoptotic ability^81–83^. Notably, when CD131 was knockdown by small interfering RNA (siRNA) transfection, the effect of EPO on endothelial cells was abolished, indicating the critical role of TPR signaling in angiogenesis^81^. EPO also significantly increased the expression of vascular endothelial growth factor (VEGF) both in endothelial cells and macrophages, which in turn stimulated angiogenesis^84,85^. In addition, lymphangiogenesis and lymph node tumor metastasis were also reported to be induced by EPO through PI3K- and ERK-dependent pathway^86^.

The interaction between EPO and anticancer agents varies in cancer type and drug mechanism. Recently, Pham et al. reported that EPO selectively modulated p53-related genes in response to genotoxic and non-genotoxic agents, thus alleviating p53-dependent apoptosis in myeloid leukemia cells^87^. The effects of EPO on non-small cell lung cancer (NSCLC) were conflicting: Merkle et al. reported that concurrent treatment with EPO decreased cisplatin-induced caspase-3, leading to less apoptosis^72^; however, Frille et al. reported that EPO did not affect cisplatin-induced apoptosis of NSCLC^73^. With regard to targeted drugs, it depended on the mechanism of the drugs. Rituximab, which functioned through complement-dependent cytotoxicity (CDC), was not affected by EPO in diffuse large B-cell lymphoma^74^. However, the anti-human epidermal growth factor receptor 2 (HER2) antibody trastuzumab was partially antagonized by concurrent treatment of EPO, which activated Jak2/Src signaling and inactivated PTEN^69^. Therefore, the application of EPO in cancer patients under anticancer treatment should be cautious.

## Advances of nonerythropoietic EPO derivatives

Due to the low affinity of TPR, a high concentration of EPO is required to exert tissue-protective and immunoregulatory effects. However, a high serum concentration of EPO could increase hematocrit levels and activate platelets and endothelia through the classical (EPOR)_2_, thereby increasing risks of thrombosis and cardiovascular events^12,88^. In a randomized trial of high-dose EPO (total dose of 100,000 IU over 3 consecutive days) in donation after cardiac death (DCD) kidney transplant patients, EPO treatment significantly increased the risk of thromboembolic events at 1 month (17.8% vs 4.3%, *P* = 0.048) and 1 year (24.4% vs 6.4%, *P* = 0.020). However, EPO groups had higher endogenous creatinine clearance at 1 year (68 ± 23 mL/min vs 57 ± 25 mL/min, *P* = 0.04). To avoid the side effects of erythropoiesis, some nonerythropoietic derivatives of EPO that only bind TPR have been developed.

### Carbamylated EPO

Carbamylated EPO (CEPO) is produced from the chemical modification of EPO. Through carbamylation of the lysine residuals, CEPO could not induce erythropoiesis but still exerted tissue-protective effects^5,8^. Similar results were obtained in other chemical mutants of EPO but all had less than optimal characteristics, including exceedingly high costs for production, structural instability and potential for antibody formation^13^. These defects limit the application of CEPO.

### Helix B surface peptide

The structure-activity relationship studies of EPO have identified regions for binding to (EPOR)_2_^9^. Interestingly, chemical modifications or mutations of these binding sites abolish erythropoiesis but retain the tissue-protective effects. This suggests that the helix B of EPO, which is exposed to aqueous medium away from the binding sites of EPO and (EPOR)_2_, is critical for the recognition of TPR. Brines confirmed that the helix B peptide had similar tissue-protective effects for EPO and CEPO but was not erythropoietic in vitro and in vivo^9^. Based on these observations, an eleven-amino acid linear peptide, QEQLERALNSS, mimicking the three-dimensional structure of the external aqueous face of the helix B peptide was developed and named helix B surface peptide (HBSP) or ARA290. Because the N-terminal residue is glutamine, HBSP undergoes a spontaneous, irreversible cyclization into pyroglutamate HBSP (pHBSP)^9^. As predicted, HBSP and pHBSP were not erythropoietic but were highly active in protecting against neural injury and ischemia-reperfusion injury (IRI) and further promoted wound healing^9^.

A number of studies have verified the protective effects of HBSP/pHBSP in different models. The immunoregulatory effects of HBSP, including suppressing chemokine and cytokine expression in monocytes/macrophages^26–28^, enhancing phagocytotic functions of macrophages^28^, promoting the development of Th2 and Treg cells but suppressing Th1 and Th17 cells^52,89^ and inducing autophagy^90,91^, have been well documented. The signaling pathway of HBSP was through the binding of TPR and activating Jak2, followed by different downstream pathways^12,16,92,93^.

### Cyclic helix B peptide

Although HBSP shows similar protective effects to EPO, a short plasma half-life of ~2 min limits its applications in the clinic^9^. Because HBSP is a linear peptide, cyclization is an efficient method to improve its metabolic stability. To prolong the half-life of HBSP, our group synthesized a series of cyclic analogs and found that the head-to-tail thioether-cyclized HBSP, namely, cyclic helix B peptide (CHBP), remained stable in human plasma and had a 2.5-folds longer half-life than HBSP in human hepatocytes. In rats, CHBP also elicited a remarkably slower metabolism in vivo^10^. Importantly, the thioether-cyclization stabilized the secondary structure of the α-helix of CHBP, making CHBP more effective in tissue protection^10,94–96^. When CHBP was applied in a preservation and reperfusion solution, it showed great protective effects in storing allografts^96^.

Proteome analysis revealed that CHBP-mediated tissue protection was tightly associated with the regulation of energy metabolism and the reduction of oxidative stress^97^. Mitochondria are the energy factories for cells and the main source of reactive oxygen species (ROS); thus, mitochondrial dysfunction underlies many diseases. Through the Nrf2 signaling pathway, CHBP reduced ROS production, alleviated endoplasmic reticulum stress and restored mitochondrial membrane potential and integrity, leading to protection against apoptosis^98,99^. In addition, CHBP suppressed the expression of transient receptor potential melastatin 7 (TRPM7), a membrane Ca^2+^ channel and kinase which was upregulated in kidney IRI, and reduced TRPM7-like current. In this way, Ca^2+^ overload-induced mitochondrial injury was also alleviated^100^.

In addition to tissue-protective effects, CHBP also modulates the activity of immune system. The Jak2/STAT3/SOCS1 signaling pathway can be activated by CHBP to inhibit the maturation of DC^38^. In a rat renal transplantation model, the decreased number of mature DCs alleviated acute renal allograft rejection^38^. For other immune cells, the effects of CHBP require further exploration.

## EPO derivatives, a promising tool of immune regulation

From erythropoiesis to immune regulation and from native EPO to nonerythropoietic derivatives, the understanding of EPO and its derivatives has been widely expanded. Generally, EPO signaling suppresses the activation of the immune system, shifts the inflammatory response to immune tolerance, protects injured tissues from apoptosis, and promotes wound healing. These effects make EPO a promising target in autoimmune diseases, allergy, IRI, and organ transplantation. Because (EPOR)_2_ and TPR are expressed on a variety of immune cells, the direct effects of EPO and its derivatives on the differentiation and function of immune cells require further study.

Notably, the development of nonerythropoietic derivatives of EPO, which eliminates the side effects of EPO, removes concerns for their application. Among them, the small peptides, including HBSP and CHBP, which mimic the three-dimensional structure of helix B, show great potential for translation to clinical drugs. A number of ongoing clinical trials on HBSP show promise (summarized in Table 1)^101–104^. HBSP has been granted Orphan Drug Designation for the treatment of sarcoidosis and the prevention of graft loss in pancreatic islet transplantation in the United States and the European Union. These peptides contain only 11 amino acids and do not undergo complicated modification, leading to lower production costs and complexity compared with traditional protein-based biopharmaceuticals. Like other star peptide drugs, such as Victoza^TM^ from Novo Nordisk and Sandostatin^TM^ from Novartis, these small peptide EPO derivatives show good specificity, efficacy and tolerability without immunogenicity^105^. Our contribution to prolonging the half-life of HBSP via thioether-cyclization, namely, CHBP, further improves the chemical and physical stability. The significant increase in half-life is more in line with clinical drug requirements with better cost effectiveness. Furthermore, innovation in alternative administration routes, such as oral preparation, with advances in drug delivery technology may provide better prospects for clinical translation and application for these biologically active peptides.

**Table 1:** Registered clinic trials of HBSP (ClinicalTrial and ICTRP, updated July 2019).

Registered no.	Start date	Status	Study contents	Conditions	Study design	Study population	Phase	Locations	Publication
EUCTR2010-018584-41-NL	July 2010	Unknown	ARA290 as therapeutic strategy in no-option critical limb ischemia patients	Critical limb ischemia	Double-blind RCT	*n* = 8	2	Netherlands	Not available
EUCTR2010-021518-45-NL	July 2010	Unknown	Effectiveness of ARA290 in the treatment of pain in neuropathic pain patients	Neuropathic pain	Double-blind RCT	Not available	Not available	Netherlands	Not available
EUCTR2010-023469-22-NL	March 2011	Unknown	ARA 290 as therapeutic strategy in rheumatoid arthritis	Rheumatoid arthritis	Non-RCT	*n* = 12	2	Netherlands	Not available
EUCTR2010-024364-18-NL	April 2011	Unknown	Effects of ARA290 on the cognitive and neural processing of emotions in healthy volunteers	Emotional information processing	Double-blind RCT	Not available	Not available	Netherlands	Not available
NTR3081	October 2011	Completed	Effectiveness of ARA290 on pain relief in sarcoidosis patients with small-fiber neuropathy	Sarcoidosis Small fiber neuropathy	Double-blind RCT	*n* = 24	Not available	Netherlands	Heij et al. [101]
NTR3131	October 2011	Recruiting	ARA290 and the ventilatory response to hypoxia and pain responses in healthy volunteers	Hypoxia; Hypoxic pulmonary vasoconstriction; Hypoxic ventilatory response	Crossover	*n* = 16	Not available	Netherlands	Not available
NCT02070783	February 2012	Completed	Effects of ARA290 on the cognitive and neural processing of emotions in healthy volunteers	Depression	Double-blind RCT	*n* = 36	1 and 2	Netherlands	Cerit et al. [102]
NTR3575	July 2012	Completed	Effects of ARA 290 on the regrowth of epidermal nerve fibers in patients with sarcoidosis	Sarcoidosis small fiber; Neuropathy pain	Double-blind RCT	*n* = 40	Not available	Netherlands	Dahan et al. [103]
EUCTR2012-003688-24-NL	December 2012	Unknown	Safety and effects of ARA 290 on pain relief in chronic pain from complex regional pain syndrome type 1	Complex regional pain syndrome	Double-blind RCT	Not available	Not available	Netherlands	Not available
NCT01933529	October 2013	Unknown	Effects of ARA290 on prediabetes and type 2 diabetes	Type 2 diabetes; Impaired glucose tolerance; Impaired fasting glucose	Double-blind RCT	*n* = 24	2	Sweden	Not available
NCT02039687	January 2014	Completed	Effects of ARA290 on corneal nerve fiber density and neuropathic symptoms of subjects with sarcoidosis	Neuropathy of Sarcoidosis	Double-blind RCT	*n* = 64	2	United States; Netherlands	Culver et al. [104]

*HBSP* helix B surface peptide, *ICTRP* International Clinical Trials Registry Platform, *RCT* randomized controlled trials.

## Conclusion

In addition to erythropoiesis and tissue-protective effects, EPO and its derivatives show great direct immune regulatory effects on immune cells. In the innate immune system, EPO and its derivatives tend to shift macrophages from M1 to M2, facilitate macrophage phagocytosis, inhibit the maturation of DCs and downregulate inflammatory reactions of mast cells. In the adaptive immune system, EPO directly suppresses lymphocytes and influences the balance of T helper cell subsets. The nonerythropoietic derivatives of EPO specifically bind TPR and regulate the activities of immune cells without increasing risks of cardiovascular complication, showing promising prospects for clinical translation. In summary, EPO derivatives are promising drugs in autoimmune diseases, allergies, organ IRI, and organ transplantation.
